# From diagnosis to treatment: patients’ attitudes toward cancer care

**DOI:** 10.3389/fpsyg.2026.1843666

**Published:** 2026-07-29

**Authors:** Ilaria Durosini, Valeria Sebri, Paolo Guiddi, Gabriella Pravettoni

**Affiliations:** 1 Department of Oncology and Hemato-Oncology, University of Milan, Milan, Italy; 2 Applied Research Division for Cognitive and Psychological Science, IEO, European Institute of Oncology IRCCS, Milan, Italy

**Keywords:** biopsychosocial model, cancer, cancer treatment, psychological support, psycho-oncology

## Abstract

A cancer diagnosis is perceived as a traumatic life event, typically characterized by an initial state of shock that involves dysfunctional emotions related to uncertainty and loss of control. Patients may experience persistent distress, anxiety, and fear of cancer recurrence, even some years after cancer treatments. Moreover, the patients’ routines are often disrupted, as are their social and intimate relationships, and they are required to reorganize their priorities and expectations. Despite these challenges, cancer may also represent for some people an opportunity for self-reflection and personal growth. In this regard, patients can activate coping resources, redefine life priorities, and develop greater self-awareness following the diagnosis. Building on this background, the present contribution describes a new conceptual psychological model that highlights patients’ attitudes toward cancer, identifying two key dimensions: *attitudes toward diagnosis* and *attitudes toward treatment*. The diagnosis may be perceived as either an obstacle or an opportunity for change; treatment, at the same time, could be either beneficial or burdensome. Therefore, four distinct psychological configurations emerged from the intersection of these two dimensions, each characterized by specific emotional, cognitive, and behavioral patterns that may influence coping strategies, treatment adherence, and identity reconstruction. To illustrate the model’s clinical relevance, the present case shows how these psychological configurations may occur in real-world oncology settings.

## Introduction

According to the most recent evidence, oncological diseases represent one of the leading causes of death worldwide. In 2022, approximately 20 million new cancer cases were reported, and an additional 1.3 million cases are projected by 2025 (IARC, 2025). Approximately one in 20 individuals will receive a cancer diagnosis over the course of their lifetime in Europe (Joint Research Centre, 2024), with relevant consequences not only on the physical domain, but also on individuals’ psychological and cognitive skills (Oliveri et al., 2019; Durosini et al., 2026). The experience of diagnosis may exacerbate a strong initial state of shock, specifically, which is often characterized by intense emotional distress, a sense of loss of control, and uncertainty (Hughes et al., 2024; Shahmari et al., 2023). Negative emotions such as distress, anxiety, and fear of cancer recurrence may arise throughout the cancer trajectory and persist over time, also in relation to external triggers (Miaskowski et al., 2020; Durosini and Pravettoni, 2023; Ongaro et al., 2021; Durosini et al., 2021; Ferrucci et al., 2020). Accordingly, the diagnosis represents a critical life event that disrupts everyday activities and previously planned life goals, affecting multiple aspects of an individual’s and intimate life (Sebri et al., 2024a; Zagami et al., 2026). Life goals previously established over the course of life may need to be postponed or left; similarly, social relationships may change or become strained, and daily activities may need to be adjusted in response to new limitations and demands imposed by the disease and its related treatments (Durosini and Pravettoni, 2023; Durosini et al., 2026). As a consequence, patients often report feelings of frustration related to uncertainty about the future (Richter et al., 2022) and difficulties returning to their previous routines (Durosini et al., 2025). Additionally, approximately 30–40% of cancer patients experience cognitive impairments, which may be exacerbated by disease progression and treatment-related side effects (Wefel et al., 2015; Lange et al., 2019). For these reasons, patients’ life-course trajectories can be complex and distressing, with the impact of past experiences and social factors shaping the cancer journey (Sahu et al., 2026). Patients report a feeling described as “lost in the loop,” a perception of fragmentation and a lack of holistic care in standardized cancer pathways (Solberg et al., 2023). In this transitional experience, the concept of *liminality* captures the cancer patients’ in-between state, where they find themselves in a state where they are neither the who they used to be not fully adapted to life as survivors (Hansen et al., 2019).

Over this healthcare journey, the psychological consequences of cancer may vary considerably depending on individuals’ psychological, social, and cognitive resources. Personal representations of the disease may be relatively independent of its clinical and objective characteristics, as they are shaped by individuals’ perceptions of illness and the meanings, they attribute to it (Durosini and Pravettoni, 2023). In line with classical theories of emotions (Frijda, 2009), predicting emotional responses remains challenging. If some patients may experience significant psychological distress in response to life events, others may demonstrate notable resilience to cope with adversities. These perceptions may affect treatment adherence, psychological well-being, and positive clinical outcomes. At the same time, supportive relationships and caregiver support are important coping resources for managing the disease’s impact on patients’ lives. Therefore, integrating a life course perspective requires capturing patients’ evolving, subjective experiences and their related meanings during this transition (Sahu et al., 2026). Hansen et al. (2018) highlighted that person-centered care integrates cancer with patients’ needs and values, aiming at tailoring psychological interventions.

In line with the aforementioned background, patients’ active participation in their healthcare pathways is crucial. Among available strategies, evidence suggests that increased self-awareness of both physical symptoms and emotional experiences can promote psychological well-being and support illness acceptance. Moreover, awareness of one’s strengths and limitations may facilitate doctor-patient relationship, collaboration, and a shared decision-making process regarding oncological pathways (e.g., Almasri and McDonald, 2023; Chichua et al., 2022). Therefore, to foster patients’ active involvement in their healthcare process, it is essential to understand how they experience illness from a psychological perspective. Additionally, fostering body awareness represents a valuable resource for attending to internal sensations and interoceptive signals, thereby supporting pain perception and management (Ekstedt and Rustøen, 2019). In this context, psychological support and interventions play a fundamental role in promoting the exploration of inner resources and coping strategies, facilitating self-discovery and enabling patients to re-evaluate life priorities (Sebri et al., 2024c) and potentially enhancing self-curiosity (Aschieri et al., 2016; Aschieri and Durosini, 2015; Aschieri et al., 2020).

## A biopsychosocial approach

In line with patients’ active participation in their care processes (Durosini et al., 2025), the biopsychosocial approach conceptualizes patients as integrated systems composed of distinct yet interrelated components that interact to support specific functions (Retroz-Marques et al., 2024). Within this framework, the experience of pain can be understood as the result of interactions among biological factors (e.g., nociceptive processes), psychological factors (e.g., pain perception and cognitive appraisal), and social factors (e.g., behavioral responses to pain; Cheatle, 2016). For example, patients’ choices are shaped by their relational context and often involve significant others (Fitzpatrick, 2016), including not only caregivers but also the broader healthcare system (Gómez-Vírseda et al., 2020). In this context, Rapley (2008), through the concept of “distributed” decision-making, and Epstein and Street (2011), with the notion of a “shared mind,” emphasized the central role of social interaction in shaping healthcare decisions (Laidsaar-Powell et al., 2017). In this regard, there are different frameworks that conceptualize social dynamics in decision-making (e.g., Laidsaar-Powell et al., 2017). For instance, the TRIO framework is an empirically-grounded conceptual framework of triadic decision-making framework that captures the multidimensional interplay among patients, family members, and healthcare professionals, highlighting a collaborative process in which decisions are jointly constructed coming from evaluating treatment options, sharing emotional experiences, and managing the disease’s physical impact (Cincidda et al., 2024). This approach can strengthen doctor-patient relationships, in which the quality of communication represents a key factor in identifying patients’ needs and comprehensively exploring their experiences. In turn, this enables the development of tailored therapeutic pathways and can promote positive clinical outcomes (Butalid, 2015; Grosso, 2025).

## Patients’ attitudes toward cancer care: an hypothesized model

Building on this perspective, the present contribution proposes a model of patients’ attitudes toward cancer care, structured along two key dimensions: *attitudes toward cancer diagnosis* and *attitudes toward oncological treatment*. In the first dimension, patients may exhibit different *attitudes toward their cancer diagnosis,* perceiving the disease either as an obstacle or as an opportunity for self-discovery and change. On the one hand, individuals who perceive the illness as an obstacle tend to focus on its detrimental personal, social, and physical consequences. Conversely, the process of self-discovery may represent an opportunity for change, as it can activate new coping resources to address illness-related issues and reframe the life trajectory toward personal growth, increasing self-awareness, and promoting the modification of previously dysfunctional aspects of daily life. Regarding *attitudes toward oncological treatment*, care may be perceived as either beneficial and enabling when patients believe in its effectiveness and in its role in supporting recovery and adaptation. Conversely, when treatment is experienced as restrictive and burdensome, patients may emphasize its negative, painful, and limiting aspects.

Based on these two dimensions, four initial patterns of response to diagnosis can be identified:

a) *Opportunity/Utility configuration:* when the illness is perceived as an opportunity and treatment as beneficial, cancer may be interpreted as a chance to disengage from excessive social demands, potentially leading to secondary gains and post-traumatic growth (Liu et al., 2020). In this configuration, treatments and their potential side effects may be accepted as part of the care process, in light of future well-being. For example, a possible sentence could be: *“This illness gave me the opportunity to re-establish my priorities. For at least twenty years, I had wanted to do something for myself without always putting others first.”*
b) *Obstacle/Utility configuration:* if the cancer is perceived as an obstacle and treatment as beneficial, the diagnosis may lead to a generative traumatic event. Patients may acknowledge the disruptive nature of the diagnosis while recognizing the need to engage in self-care and adhere to the prescribed treatment pathway. Similarly, patients could be more prone to accept potential side effects to promote well-being. In this regard, patients could report reflections as: “*This recurrence gives me the opportunity to re-evaluate how I have lived all these years. When I was young, I had a great body, but after pregnancy, I did not take care of myself as much. Now I want to feel beautiful again, to rediscover that carefree, teenage part of myself that I left behind too many years ago.*”
c) *Opportunity/Restrictive configuration:* if the cancer is perceived as an opportunity and treatment as restrictive, it may be experienced as a constraint. Specifically, patients may attribute one or more meanings to the experience of illness, viewing it as an opportunity to redefine life priorities and goals in pursuit of a more integrated sense of identity. However, treatment may be perceived as limiting, diverting attention from one’s personal path as patients could report in this way: “*At last, I do not care if this hole (abdominal fistula) closes. It reminds me of my sister, who was alive the last time I was hospitalised here, and that makes me feel good, because it gives me the illusion that she is still at home waiting for me.*”
d) *Obstacle/Restrictive configuration:* if the cancer is perceived as an obstacle and treatment as restrictive, the diagnosis may be a disruption of personal and social roles, characterized by a sense of loss and a breakdown of one’s previous identity (Gökler-Danışman et al., 2017). Therefore, the individual’s system of values and beliefs must be considered throughout the care pathway, alongside the relationship with the healthcare team, as expressed in this example sentence: “*I am very angry about this diagnosis. I did not want this disease; I have lived a healthy lifestyle my whole life to avoid it. And now I do not see why I should get treatment. Both my mother and my mother-in-law died of the same disease; one sought treatment, and the other did not. And yet they both died.*”

### How to manage psychological configurations?

**Figure 1:**
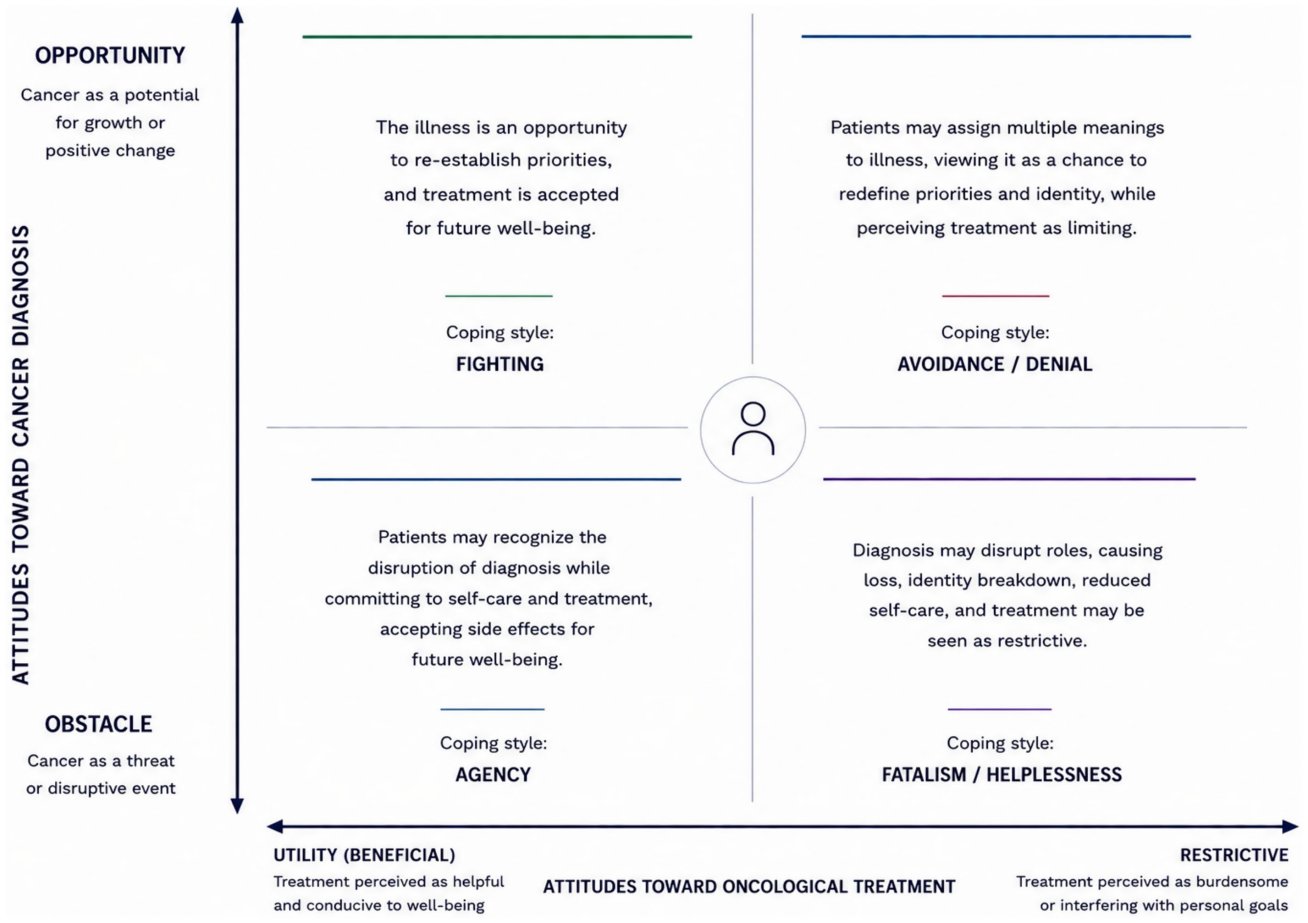
A conceptual framework of patients’ attitudes toward the cancer care.

Psychological support can help patients view the cancer experience as an opportunity to re-establish life priorities aligned with their personal values and goals. Considering the different psychological configurations proposed by the model, it is important to identify the key domains to be addressed and the corresponding targeted psychological interventions (Semenenko et al., 2023), as outlined below (see Figure 1):

1) *Opportunity/Utility configuration*: Patients within this framework tend to demonstrate effective self-care abilities; psychological interventions should focus on reinforcing personal resources, building on the strengths that people already exhibit. In particular, it is important to explore the meaning patients attribute to the illness and their motivations for facing it, to support the maintenance of self-awareness and resilience. Accordingly, the main coping strategy is *fighting*, in terms of coping with physical and psychological issues, thanks to personal resources that reinforce resilience.
2) *Obstacle/Utility configuration*: Patients within this framework are often strongly affected by the diagnosis as a traumatic event; psychological interventions should focus on addressing illness-related meanings and patients’ potentially maladaptive approaches to the illness. In line with the Acceptance and Commitment Therapy (ACT) framework, it could be useful to promote patents’ psychological flexibility, helping them to re-evaluate maladaptive thoughts without allowing these to interfere with daily functioning (Li et al., 2021; Graham et al., 2024). This way, *agency* is the main coping strategy as focused on the perception of control to handle illness difficulties effectively, regulating thoughts and behaviors in an appropriate way.
3) *Opportunity/Restrictive configuration*: Within this framework, patients may experience difficulties in managing oncological treatments and their related side effects; psychological interventions should focus on fostering cancer-related consequences’ acceptance, addressing both physical and psychological aspects. At the same time, identifying functional coping strategies to support treatment adherence is crucial. In this regard, reducing maladaptive thought patterns through relaxation techniques may be beneficial, thereby promoting a shift in attention toward more adaptive, present-focused experiences (e.g., Mindfulness-Based Cognitive Therapy; Hughes et al., 2017). Therefore, relation techniques could be supported by the *avoidance/denial* coping strategy mainly, allowing for disengagement from the stressors to focus on resources.
4) *Obstacle/Restrictive configuration*: Patients within this framework may experience difficulties both in accepting the illness and in managing treatment-related side effects; therefore, psychological interventions should aim to restore a sense of self beyond the illness, emphasizing the individual as a “person” rather than solely as a “patient.” In this regard, interventions aimed at reducing stress and enhancing self-awareness may facilitate the identification and management of underlying sources of stress (e.g., Cognitive Behavioral Stress Management; Tang et al., 2020). In this configuration, *fatalism/helplessness* is a crucial coping strategy because it helps patients to accept the perception of loss of control, reducing the risk of trying to solve illness issues.

## The case of Susan

### Clinical case overview

Susan is a 54-year-old Italian woman, married to Marc for 15 years, and the mother of a 20-year-old daughter. She lives in the Italian countryside, in a farmhouse divided into three apartments, where a couple of close friends also reside. Susan describes herself as physically active, autonomous, and highly independent. She regularly played tennis and participated in triathlons. Throughout her life, she has devoted considerable energy to her professional career. She completed her studies in graphic design with excellent results and subsequently began working at a major Italian company, where she developed strong professional skills and attained a prominent role. When reflecting on her childhood, Susan reported a long-standing and emotionally demanding relationship with her mother, which contributed to feelings of not being fully recognized or valued within her family. In contrast, she describes a very positive relationship with her father, whom she considered an important source of support and guidance. Susan’s father passed away in early 2019, and a few months later she received a diagnosis of stage I breast cancer, without lymph node involvement. From a clinical perspective, she underwent surgical treatment consisting of a radioguided quadrantectomy of the right breast with axillary sentinel lymph node biopsy.

**Figure 2:**
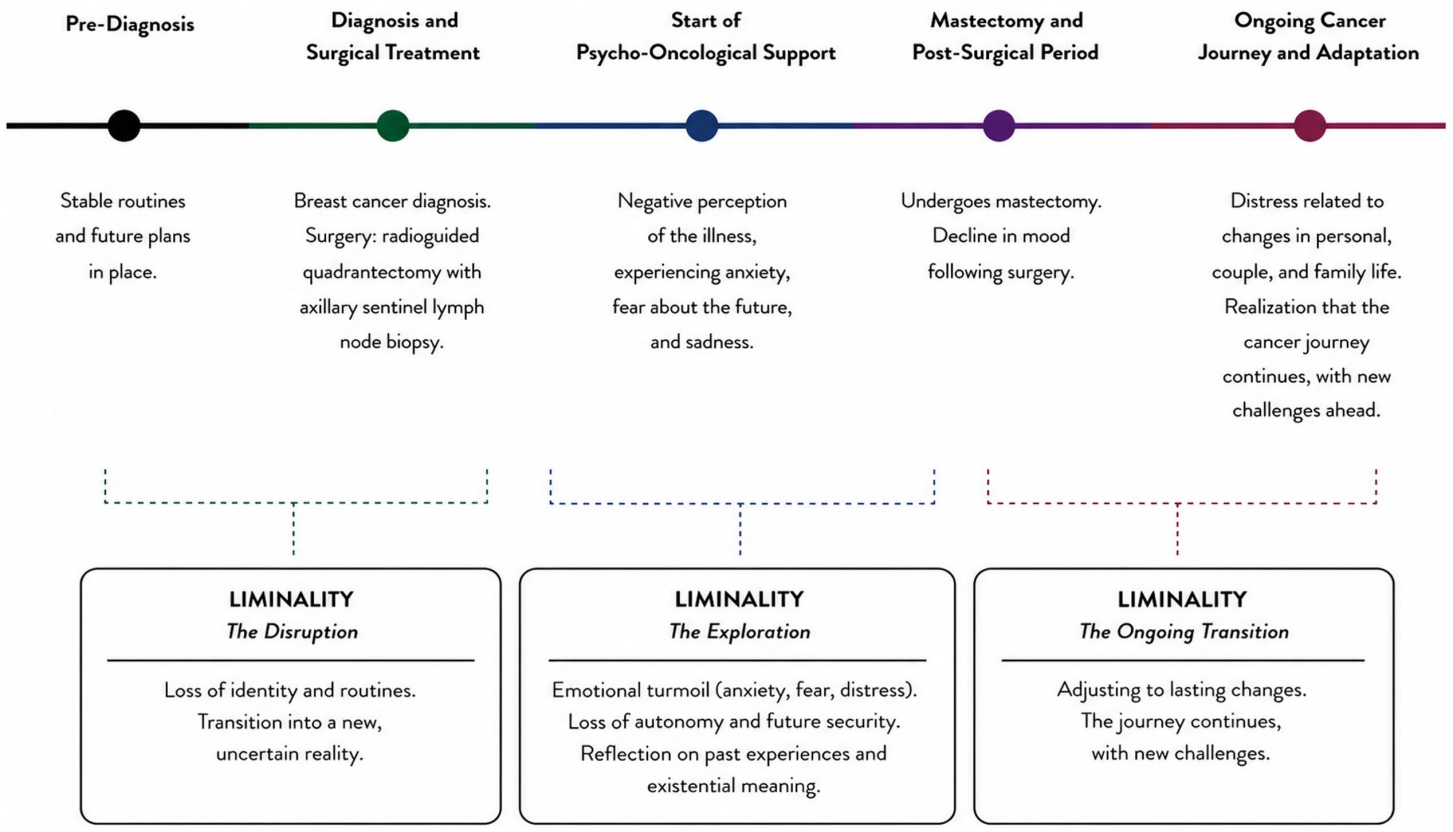
Trajectory of liminality across Susan’s breast cancer experience.

The cancer diagnosis represented a profound disruption in Susan’s life, significantly impacting her daily activities and interrupting established routines. In line with the theoretical background, this shows the first time Susan experiences *liminality* - a phase where she no longer recognizes herself pre-illness, disrupting usual habits and forcing her to approach a new way of living. Consequently, she began psycho-oncological support in February 2020. Initial psychological sessions revealed that Susan predominantly interpreted the illness in negative terms, perceiving it as an obstacle to her life trajectory and experiencing intense anxiety, fear about the future, and sadness. Early consultations, therefore, focused on managing anxiety related to the diagnosis and uncertainty about the future, as well as concerns that treatment side effects might compromise her independence and autonomy. She also reported previous distressful life events, noting that the diagnosis reactivated painful memories, particularly those related to caring for her father in his final years. Additionally, she expressed fears about not being able to raise her daughter and “not being there for her graduation and wedding.” In this second experience of *liminality*, dysfunctional emotions emerged, primarily as anxiety and distress. This phase is characterized by a perceived loss of autonomy and the fear of the future, which prompts existential questions. Additionally, the recollection of painful memories represents another core feature of *liminality*, forcing a reflection on how past experiences are intrinsically linked to the present. Lastly, following the mastectomy, which was associated with a decline in mood, Susan reported significant distress related to the changes in her personal, couple, and family life since the operation. This marks the third episode of *liminality* following the surgery, a phase where Susan realizes that while the mastectomy is behind her, the overall cancer journey is far from over as she dealing with new changes and challenges. Figure 2 illustrates Susan’s trajectory of liminality across the breast cancer experience.

### Psychological configuration of illness

Based on the proposed psychological configurations, Susan can be categorized within the *Obstacle/Utility configuration*. In line with this framework, initial interventions focused on addressing dysfunctional emotional responses. Several strategies were introduced to help Susan better manage her condition. In particular, cognitive restructuring techniques were implemented to promote psychological flexibility, enabling her to acknowledge maladaptive thoughts without allowing them to interfere with daily functioning.

Psychological sessions also supported Susan in recognizing the need to engage in self-care, thereby fostering adherence to treatment despite the presence of side effects, such as frequent paraesthesia, vaginal dryness, and cognitive difficulties (e.g., “chemo brain”). Moreover, these interventions fostered psychological flexibility, enabling her to better manage treatment-related side effects in daily activities. This process allowed Susan to identify and mobilize her coping skills (i.e., personal, dyadic, family, and work-related resources) in order to navigate her cancer journey more effectively. One year after the initial diagnosis, during ongoing oncological treatments, fatigue and fears related to changes in self-image became more prominent. In addition, work interruption contributed to a perceived loss of autonomy and increased psychological distress. Susan also expressed significant concern about persistent pain, which was almost constant and substantially limited her daily activities. Consequently, psychological support focused on helping her readjust the discrepancy between her current self-image (*“I feel like I’m seventy”*) and her pre-illness identity as an active and athletic woman. At this stage of the illness, an *Obstacle/Restrictive configuration* emerged. Accordingly, the psycho-oncologist introduced an intervention aimed at reducing stress, enhancing self-awareness of stressors, and strengthening the patient’s sense of agency. This approach enables the use of techniques to manage distressing symptoms, thereby improving the patient’s perceived control over her life and overall functioning.

The end of oncological treatments also represented an opportunity to reflect on the long-term psychological impact of the illness. Psychological sessions focused on the patient’s need to feel loved and accepted in her needs, consistent with an *Opportunity/Utility configuration*. Additionally, sessions addressed physical changes affecting sexuality, leading Susan to initiate couple-based psychological counseling aimed at improving communication and relational well-being. As this new focus emerged, the patient chose to gradually disengage from psycho-oncological support and continue with couple therapy outside the clinical setting. In conclusion, Susan reported that the illness allowed her to gain a “secondary benefit” by making her needs more visible to the people she loves. Her cancer experience thus became an opportunity for greater self-understanding, enabling her to express her needs and desires more openly, without fear of upsetting her loved ones.

## Discussion

The present contribution proposes an innovative framework of psychological configurations to address emotional and cognitive challenges among cancer patients, offering tailored interventions based on individual patient characteristics. In line with a patient-centered point of view, addressing patients’ needs involves not only managing physical symptoms but also supporting them in identifying and mobilizing coping resources across multiple domains of life, including personal, relational, and social contexts (Kasgri et al., 2024). At the same time, exploring and understanding patients’ expectations is crucial to support a shared approach to care, improve treatment adherence, and increase their perceived quality of care (Cockle and Ogden, 2022; Sebri et al., 2024b). Within the present context, psychological configurations should not be considered static; rather, individuals may shift between configurations over time, depending on different factors (e.g., personality traits, disease trajectories, and personal values and needs). In this context, supporting patients in reframing their illness experience as a potential turning point remains a key challenge in psychological care. For this aim, psychologists must recognize that the evolving configuration is a key task throughout the care journey, designing and implementing intervention strategies tailored to patients’ needs and adapting their approaches to each individual’s unique characteristics within a shared decision-making process (Chichua et al., 2025; Sebri et al., 2025). In accordance with the literature, cancer can represent an opportunity to re-evaluate life goals and pursue more meaningful directions. It is therefore crucial to align theoretical approaches and perspectives within clinical practice, following a patient-centered approach (Bilodeau et al., 2022). In this regard, promoting meaningful relationships with healthcare professionals, as reliable guides in coping with uncertainty, can enhance effective illness management (Sebri et al., 2024d). In conclusion, patients can position themselves at the center of their lives, considering their values and shifting from a learned helplessness approach to one of hopefulness. In this way, psycho-oncological support can foster new strategies of thinking to facilitate cancer acceptance, accepting what has occurred, and integrating illness-related changes into the self. This way, patients could have the possibility to transform illness from an unchosen, critical event into a meaningful and integrated part of their biography.

## Conclusion

The present article proposes four specific configurations to explore patients’ characteristics and attitudes across different phases of treatment, thereby informing the development of tailored psychological interventions. Psychological support plays a fundamental role in empowering patients and fostering their active participation in the care process, particularly within a biopsychosocial framework that addresses not only the patient but the person as a whole. From this perspective, healthcare professionals are called to consider the full spectrum of well-being, adopting strategies tailored to each individual.

## Data Availability

The original contributions presented in the study are included in the article/supplementary material, further inquiries can be directed to the corresponding author.
